# Healthy lifestyle counteracts the risk effect of genetic factors on incident gout: a large population-based longitudinal study

**DOI:** 10.1186/s12916-022-02341-0

**Published:** 2022-04-29

**Authors:** Yuan Zhang, Rongrong Yang, Abigail Dove, Xuerui Li, Hongxi Yang, Shu Li, Ju Wang, Wei-Dong Li, Hongyu Zhao, Weili Xu, Yaogang Wang

**Affiliations:** 1 School of Public Health, Tianjin Medical University, Qixiangtai Road 22, Heping District, 300070 Tianjin, China; 2 Public Health Science and Engineering College, Tianjin University of Traditional Chinese Medicine, Tianjin, China; 3 Aging Research Center, Department of Neurobiology, Health Care Sciences and Society Karolinska Institutet and Stockholm University, Stockholm, Sweden; 4 Department of Epidemiology and Biostatistics, School of Public Health, Tianjin Medical University, Tianjin, China; 5 School of Management, Tianjin University of Traditional Chinese Medicine, Tianjin, China; 6 School of Biomedical Engineering and Technology, Tianjin Medical University, Tianjin, China; 7 Department of Genetics, School of Basic Medical Sciences, Tianjin Medical University, Tianjin, China; 8 Department of Biostatistics, Yale School of Public Health, Yale University, New Haven, USA

**Keywords:** Polygenic risk, Healthy lifestyle, Gout, Prospective study, UK Biobank

## Abstract

**Background:**

Risk genes linked to the development of gout have been identified, and lifestyle factors are related to gout risk. It remains unclear whether healthy lifestyle factors can mitigate the genetic risk of gout. Therefore, we aimed to explore whether and to what extent a healthy lifestyle can mitigate the risk of gout related to genetic factors.

**Methods:**

Within the UK Biobank, 416,481 gout-free participants (aged 37–74) were identified at baseline. Polygenic risk for gout was assessed and categorized as low (lowest tertile), middle (tertile 2), and high (highest tertile). Healthy lifestyle factors included no/moderate alcohol consumption, no smoking, physical activity, and a healthy diet. Participants were categorized into three groups according to their number of healthy lifestyle factors: unfavorable (0 or 1), intermediate (any 2), and favorable (3 or 4). Data were analyzed using Cox proportional hazard models.

**Results:**

Over the follow-up (median: 12.1 years), 6206 participants developed gout. Compared to low genetic risk, the hazard ratios (HRs) and 95% confidence intervals (CIs) of gout was 1.44 (1.35–1.54) for middle and 1.77 (1.66–1.89) for high genetic risk. The HRs (95% CIs) of gout were 0.63 (0.59–0.67) for a favorable lifestyle and 0.79 (0.75–0.85) for an intermediate lifestyle, compared to an unfavorable lifestyle. In joint effect analysis, compared to participants with low genetic predisposition and a favorable lifestyle, the HRs (95% CIs) of gout were 2.39 (2.12–2.70)/3.12 (2.79–3.52) in those with middle and high genetic predisposition plus unfavorable lifestyle profiles, and 1.53 (1.35–1.74)/1.98 (1.75–2.24) for those with middle and high genetic predisposition plus favorable lifestyle profiles, respectively. Moreover, compared to an unfavorable lifestyle, the HRs of gout related to a favorable lifestyle was 0.64 (95% CI, 0.56–0.73) for low genetic risk, 0.65 (95% CI, 0.58–0.72) for middle genetic risk, and 0.62 (95% CI, 0.57–0.69) for high genetic risk. There was a significant additive interaction between unfavorable lifestyle and high genetic risk on gout.

**Conclusions:**

Healthy lifestyle was associated with a lower risk of gout and may attenuate the risk of gout related to genetic factors by almost a third.

**Supplementary Information:**

The online version contains supplementary material available at 10.1186/s12916-022-02341-0.

## Background

Gout is caused by deposition of monosodium urate crystals within joints after longstanding hyperuricemia [1]. It is the most common form of inflammatory arthritis, with prevalence ranging from 1 to 4% in Europe [2], 3.9% in the USA [3], and <1–6.8% globally [4–6]. Both the prevalence and incidence of gout have increased steadily in recent years, particularly in the UK [2]. In addition to causing excruciating arthritic pain, gout is associated with cardiovascular and renal diseases and is an important risk factor for premature mortality [7–9]. Despite advances in understanding the pathophysiology of gout and increasing availability of effective urate-lowering therapies, clinical management of gout continues to be suboptimal [10, 11]. Therefore, identifying individuals at high risk of developing gout may contribute to the prevention and early treatment of gout and its subsequent complications.

It is well-accepted that both genetic and lifestyle factors play a role in determining an individual’s risk of gout. There is increasing evidence that the genetic basis of gout is polygenic [12]. Genome-wide association studies (GWASs) have been successful in identifying genetic variants that are associated with hyperuricemia or gout, including *SLC2A9*, *ABCG2*, *GCKR*, and *SLC22A11* [13–16]. Gout is highly heritable (approximately 30%), and studies have identified over 20 disease-associated genomic loci [17, 18]. Polygenetic risk scores that aggregate multiple risk alleles could provide a continuous and quantitative measure of genetic susceptibility for gout.

Accumulating evidence suggests that lifestyle factors including alcohol consumption, smoking, diet, physical activity, and body mass index are associated with gout risk [19–24]. Alcohol consumption has been confirmed as a potential risk factor for hyperuricemia and is considered a trigger for gout attacks [19]. Higher intake of seafood, red and processed meats, and refined grains may increase gout risk by raising serum uric acid concentrations [25]. Moreover, the risk of gout is lower in men who are more physically active and maintain the ideal body weight based on male runners [22]. Many of the aforementioned lifestyle factors tend to cluster together and could represent interacted pathophysiological pathways underlying gout development. However, the effect of an overall healthy lifestyle on the risk of gout due to genetic factors remains unknown. In the present study, we aimed to (1) examine the association of genetic and lifestyle factors with the risk of gout and (2) explore whether a favorable lifestyle can mitigate the risk of gout related to genetic factors.

## Methods

### Study design and population

The UK Biobank is comprised of data from a population-based cohort study that recruited more than 500,000 participants (aged 40–79 years) who attended one of 22 assessment centers across the UK between 2006 and 2010 and were followed up to 2021 [26]. The present analyses were restricted to individuals who were of white British ancestry because they had available genetic information. Among 502,507 total participants, 84,993 were excluded in this cohort study, including 3124 with prevalent gout, 29,640 of non-British descent, 13,416 with missing data on genetic risk, 38,113 with missing data on any of the lifestyle factors (i.e., alcohol consumption, smoking status, physical activity, and diet) at baseline, and 1033 who were lost to follow-up examinations. This left 416,481 participants remaining for the current study (Additional file 1: Fig. S1).

UK Biobank has ethics approval from the North West Multi-Centre Research Ethics Committee (11/NW/0382). Appropriate informed consent was obtained from participants and ethical approval was covered by the UK Biobank. This research has been conducted using the UK Biobank Resource under the project number of 45676.

### Data collection

Information on sex, age, education, socioeconomic status, and employment status was collected through a touchscreen questionnaire and interview, and BMI was obtained from physical measurement. Education was defined as college or university, upper secondary, lower secondary, vocational, or other. Socioeconomic status was defined based on the Townsend deprivation index [27] (encompassing information on social class, employment, car availability, housing) and categorized as low (highest quintile), middle (quintiles 2 to 4), or high (lowest quintile) [28]. Employment status was categorized as working, unemployed, retired, or other. BMI was calculated as weight (kg) divided by height squared (m^2^) and was categorized as <25 kg/m^2^ and ≥25 kg/m^2^. Information on urate, C-reactive protein, serum creatinine, cholesterol, and triglyceride levels was obtained from blood samples collected at study recruitment.

Information on disease history was derived from medical examinations, self-reported medical conditions, and hospital inpatient records, including data on admissions and diagnoses from the Hospital Episode Statistics in England (dating back to 1997), the Scottish Morbidity Record (dating back to 1981), and the Patient Episode Database in Wales (dating back to 1998). Information on death was obtained through linkage to national death registries from May 2006 to February 2021, and the main cause of death for each participant was identified based on International Classification of Diseases 10 (ICD-10) codes.

In the UK Biobank, genotyping was performed by Affymetrix on two arrays, namely the UK BiLEVE Axiom array and UK Biobank Axiom array. Genotypes were imputed using computationally efficient methods combined with the Haplotype Reference Consortium and UK10K haplotype resource. Genetic analysis in this study was conducted using version 3 of the UK Biobank imputed data, which was released in March 2018. Our genotyping data were restricted to 13.7 million single nucleotide polymorphisms (SNPs) following the Neal-lab-performed variant quality control filters (https://github.com/Nealelab/UK_Biobank_GWAS).

### Assessment of gout

Gout was ascertained based on information from self-report (Data-Field 20002, code: 1466), medical records (ICD-10 codes: M10.0, M10.2, M10.3, M10.4, M10.9), and death records (ICD-10 codes: M10.0, M10.2, M10.3, M10.4, M10.9) from the Hospital Episode Statistics (England), the Scottish Morbidity Record (Scotland), and the Patient Episode Database (Wales).

### Assessment of lifestyle factors

Information on alcohol consumption, smoking status, and physical activity was obtained from the touchscreen questionnaire; diet was derived from the Food Frequency Questionnaire. Alcohol consumption was calculated based on self-reported intake of red wine, white wine, beer, spirits, and fortified wine. No/moderate alcohol consumption was defined as 0 to 14 g/day alcohol for women and 0 to 28 g/day alcohol for men [29]. Smoking status was dichotomized as smoking vs. non-smoking. Physical activity was measured as minutes per week spent walking or engaged in moderate or vigorous activity according to the International Physical Activity Questionnaire (IPAQ). Regular physical activity was defined as engaging in moderate activity ≥150 min per week, vigorous activity ≥75 min per week, or moderate and vigorous activity ≥150 min/week [30]. A healthy diet score was generated based on the seven commonly eaten food groups following a more recent definition of ideal intake of dietary components for cardiometabolic health [31]. A healthy diet was based on intake of at least four of these seven commonly eaten food groups [31]. Additional file 1: Table S1 provides additional details regarding the assessment of healthy lifestyle factors.

In the current study, we confirmed four healthy lifestyle factors including no/moderate alcohol consumption, not smoking, regular physical activity, and a healthy diet. Participants were categorized into three groups according to the number of healthy lifestyle factors: (1) unfavorable (0 or 1 healthy lifestyle factors), (2) intermediate (2 factors), and (3) favorable (3 or 4 factors).

### Assessment of polygenic risk score

A weighted polygenetic risk score (GRS) for gout was calculated to assess the cumulative effect of genetic risk on gout. Using an LD clumping cut-off of *r*^2^ < 0.01 and conditional analyses, we selected 33 independent SNPs that have been previously associated with gout in a Global Urate Genetics Consortium study of individuals of European descent [18]. The GRS for gout disease is based on genome-wide association studies of individuals of European descent [18]. Therefore, the present study was restricted to individuals whose self-reported ethnic background was white. Details regarding the selected SNPs are provided in Additional file 1: Table S2. Briefly, we summed the number of risk alleles (0, 1, or 2) for each SNP weighted by the effect size (β coefficient) between that SNP and gout from previous GWAS studies (Additional file 1: Methods). An individual-level genetic risk score (GRS) was then derived from the sum of the number of risk alleles present at each SNP weighted by the effect sizes from all SNPs included in the UK Biobank, which was produced using the PLINK “–score” command. Participants were divided into low (lowest tertile), middle (tertile 2), and high (highest tertile) genetic risk categories according to their Z-standardized GRS.

### Assessment of cardiometabolic diseases

Cardiometabolic diseases (CMDs) were defined as cardiovascular disease, hypertension, and/or type 2 diabetes. Information on cardiovascular disease status (i.e., presence of coronary heart disease, heart failure, atrial fibrillation, or stroke) was derived from medical records (ICD-10 codes I20-I25, I42, I48, I50, I60-I64, G45, G46). Diabetes was ascertained on the basis of medical records (ICD-10 codes E111), glycated hemoglobin ≥6.5%, and use of anti-diabetic drugs. Hypertension was defined as systolic blood pressure (SBP) ≥140 mmHg or diastolic blood pressure (DBP) ≥90 mmHg, use of anti-hypertension agents, or medical records (ICD-10 codes I10-I13, I15).

### Statistical analyses

Baseline characteristics of the study population were compared by incident gout status using *t* tests for continuous variables and chi-squared tests for categorical variables. If continuous variables did not follow a normal distribution, the Mann–Whitney *U* test was applied. Incidence rates (IRs) and 95% confidence intervals (95% CIs) per 1000 person-years were calculated for each genetic predisposition and lifestyle profile category.

Cox proportional hazards regression models were applied to estimate the hazard ratios (HRs) and 95% CIs of gout in relation to genetic risk and lifestyle factors. Follow-up year was used as the time scale in the model. Follow-up time was calculated as the time from baseline assessment until the first event of gout, death, or February 31, 2021, whichever occurred first. The proportional hazards assumptions for the Cox model were tested using the Schoenfeld residuals method, and no violations of the assumption were observed. Models were adjusted for sex, age, socioeconomic status, education level, cardiovascular disease, diabetes, hypertension, and concentrations of C-reactive protein, serum creatinine, cholesterol, triglycerides, and BMI. If data was missing for a covariate, we used multiple imputations based on five replications and utilized a chained-equation method to account for the missing data [32]. The combined effect of the lifestyle profile and genetic predisposition on gout disease risk was assessed by creating dummy variables based on the joint exposures to both factors. The presence of an additive interaction was examined by estimating the relative excess risk due to interaction (RERI), the attributable proportion (AP), and the synergy index (SI). Additionally, we examined the multiplicative interaction between lifestyle and genetic predisposition by incorporating the two variables and their cross-product term in the same model. Furthermore, we conducted stratification analysis by CMD status (with vs without) to investigate whether the associations between gout and the joint exposures of lifestyle profile and genetic predisposition varied by CMD status.

Several additional analyses were performed to assess the robustness of our study results. First, we conducted a weighted healthy lifestyle score based on the β coefficients of each lifestyle factor in the Cox proportional hazards regression model adjusted for sex, age, socioeconomic status, education level, C-reactive protein, serum creatinine, cholesterol, triglyceride, cardiovascular disease, diabetes, hypertension, and BMI. Weighted lifestyle score = (β_1_*factor 1+β_2_*factor 2+β_3_*factor 3+β_4_* factor 4) [4/(β_1_+ β_2_ + β_3_ + β_4_ )] [33]. This weighted score ranges from 0 to 4 points. Lifestyle was recorded as “favorable” (3 or 4 points), “intermediate” (2 points), and “unfavorable” (0 or 1 points). Next, we used stratification analysis to examine whether the association between gout and the joint exposures of lifestyle profile and genetic predisposition varied by age (<60 vs. ≥60 years) or by sex. Additionally, to address the role of potential reverse causality, we repeated the main analyses in a sample excluding participants who developed incident gout in the first 3-year follow-up period and participants who died within 3 years from baseline. Furthermore, we excluded participants with diuretic antihypertensive drugs at baseline to assess the robustness of our study results. Finally, we assessed the competing risk of non-gout death on the association between gout and the joint exposures of lifestyle and genetic predisposition using the subdistribution method proposed by Fine and Grey [34].

All analyses were performed using STATA 15 statistical software (Stata Corp, College Station, TX, USA) and R (version 3.6.1, R Foundation for Statistical Computing). All *P* values were two-sided, and statistical significance was set at 0.05.

## Results

### Baseline characteristics of the study population

Of the 416,481 study participants, 191,234 (45.9%) were men, and the mean (SD) age was 56.6 (8.0) years. Over a median of 12.1 years (4,921,809 person-years) of follow-up, there were 6206 cases of incident gout (prevalence = 1.5%). Compared to the gout-free participants, those who developed incident gout were more likely to be older, male, excessive alcohol drinkers, smokers, or physically inactive and to have an unhealthy diet, CMDs, lower educational attainment and socioeconomic status, and high levels of C-reactive protein, serum creatinine, cholesterol, and triglycerides (Table 1).

**Table 1:** Baseline characteristics of participants by incident gout (*N*=416,481)

Characteristic	Incident gout		*P* value^a^
	Yes (*n*=6206)	No (*n*=410,275)	
**Demographic factors**			
Age, mean (SD), year	60.86 (6.74)	56.56 (8.05)	<0.001
Male, n (%)	5243 (84.5)	185,991 (45.3)	<0.001
Education level, *n* (%)			<0.001
College or University	1453 (23.4)	138,266 (33.7)	
Upper secondary	516 (8.3)	48,187 (11.7)	
Lower secondary	1550 (25.0)	111,725 (27.2)	
Vocational	647 (10.4)	26,546 (6.5)	
Other	2040 (32.9)	85,551 (20.9)	
Socioeconomic status, *n* (%)			<0.001
High	1175 (18.9)	88,390 (21.5)	
Middle	3680 (59.3)	250,837 (61.1)	
Low	1351 (21.8)	71,048 (17.3)	
Current employment, *n* (%)			0.201
Worked	3603 (58.1)	234,324 (57.1)	
Retired	1987 (32.0)	136,580 (33.3)	
Unemployed	104 (1.7)	6716 (1.6)	
Other	512 (8.3)	32,655 (8.0)	
**Lifestyle factors**			
Alcohol consumption, *n* (%)			
Excessive	3144 (50.7)	161,796 (39.4)	<0.001
Never/moderate	3062 (49.3)	248,479 (60.6)	
Smoking status, *n* (%)			<0.001
Smoker	3768 (60.7)	186,502 (45.5)	
Non-smoker	2438 (39.3)	223,773 (54.5)	
Physical activity, *n* (%)			
Inactive	2986 (48)	173,056 (42.2)	<0.001
Active	3220 (52.0)	237,219 (57.8)	
Diet, *n* (%)^b^			<0.001
Unhealth	3312 (53.3)	160,991 (39.2)	
Health	2894 (46.7)	249,284 (60.8)	
Lifestyle index^c^			<0.001
Favorable	1726 (27.8)	184,978 (45.1)	
Intermediate	2145 (34.6)	134,948 (32.9)	
Unfavorable	2335 (37.6)	90,349 (22.0)	
Body mass index, *n* (%)			<0.001
≥25 kg/m^2^	2952 (47.6)	94,688 (23.1)	
<25 kg/m^2^	3254 (52.4)	315,587 (76.9)	
**Cardiometabolic diseases**			
Cardiovascular disease^d^			<0.001
No	4269 (68.8)	369,200 (90)	
Yes	1937 (31.2)	41,075 (10.0)	
Diabetes			<0.001
No	5383 (86.7)	390,369 (95.1)	
Yes	823 (13.3)	19,906 (4.9)	
Hypertension			<0.001
No	2296 (37)	253,941 (61.9)	
Yes	3910 (63.0)	156,334 (38.1)	
**Biomarkers**			
Urate (umol/L)	416.73 (100.25)	306.65 (78.10)	<0.001
C-reactive protein (mg/L)	3.76 (5.44)	2.52 (4.14)	<0.001
Serum creatinine (umol/L)	87.85 (37.92)	71.99 (16.27)	<0.001
Cholesterol (mmol/L)	5.34 (1.19)	5.72 (1.11)	<0.001
Triglyceride (mmol/L)	2.36 (1.33)	1.73 (0.98)	<0.001
**Genetic risk**^e^			<0.001
Low	1423 (22.9)	143,437 (35.0)	
Middle	2165 (34.9)	138,885 (33.9)	
High	2616 (42.2)	127,953 (31.2)	

^a^*P* value was calculated by comparing the baseline characteristics between the gout-free participants and participants who developed gout

^b^Diet pattern included seven dietary components: fruits, vegetables, whole grains, refined grains, fish, unprocessed meat, and processed meat

^c^Lifestyle index was created by four healthy lifestyle factors: never/moderate alcohol consumption, no smoking, regular physical activity, and a healthy diet. Participants were categorized into three groups according to the number of healthy lifestyle factors: (1) unfavorable (0 or 1), (2) intermediate (any 2), and (3) favorable (3 or 4)

^d^Cardiovascular disease included coronary artery disease, heart failure, atrial fibrillation, and stroke

^e^Weighted polygenetic risk score of gout was generated by 33 single nucleotide polymorphisms. Genetic risk categories defined according to the polygenic risk score as low (lowest tertile), middle (tertile 2), and high (highest tertile)

### Association between gout and genetic risk and lifestyle factors

In multi-adjusted Cox regression models, the HRs and 95% CIs of gout were 1.44 (1.35–1.54) for participants with a middle genetic predisposition and 1.77 (1.66–1.89) for those with a high genetic predisposition, compared to those with a low genetic predisposition (Table 2). The risk of incident gout was higher for participants with a high vs. low genetic predisposition (Additional file 1: Fig. S2a).

**Table 2:** Basic- and multi-adjusted hazards ratios (HRs) and 95% confidence intervals (CIs) of gout by lifestyle factors and genetic risks: results from Cox regression models

Factors	No. of event	IR (95% CI)^a^	Basic-adjusted HR (95% CI)^b^	Multi-adjusted HR (95% CI)^c^
**Genetic risk**				
Low	1423	0.83 (0.79–0.87)	1.00 (Ref.)	1.00 (Ref.)
Middle	2165	1.30 (1.25–1.36)	1.40 (1.31–1.50)	1.44 (1.35–1.54)
High	2618	1.70 (1.63–1.76)	1.70 (1.59–1.81)	1.77 (1.66–1.89)
**Lifestyle factors**				
Alcohol consumption				
Excessive	3144	1.61 (1.56–1.67)	1.00 (Ref.)	1.00 (Ref.)
Never/moderate	3062	1.03 (0.99–1.07)	0.64 (0.61–0.68)	0.67 (0.63–0.70)
Smoking status				
Smoker	3768	1.70 (1.64–1.75)	1.00 (Ref.)	1.00 (Ref.)
Non-smoker	2438	0.90 (0.87–0.94)	0.81 (0.76–0.85)	0.89 (0.84–0.93)
Physical activity				
Inactive	2977	1.44 (1.38–1.49)	1.00 (Ref.)	1.00 (Ref.)
Active	3229	1.13 (1.10–1.17)	0.84 (0.80–0.88)	0.90 (0.86–0.95)
Diet				
Unhealthy	3303	1.71 (1.65–1.77)	1.00 (Ref.)	1.00 (Ref.)
Healthy	2903	0.97 (0.94–1.00)	0.85 (0.81–0.90)	0.90 (0.85–0.94)

^a^Incidence rates are provided per 1000 person-years

^b^Adjusted for sex and age

^c^Adjusted for sex, age, socioeconomic status, education level, C-reactive protein, serum creatinine, cholesterol, triglyceride, cardiovascular disease, diabetes, hypertension, genetic risk, each lifestyle factor, and body mass index (BMI)

*Abbreviations*: *IR* incidence rate

In multi-adjusted Cox regression models, each of the four lifestyle factors we examined was associated with a lower risk of gout. Specifically, no/moderate alcohol consumption, not smoking, regular physical activity, and having a healthy diet were associated with a 33% (HR, 0.67; 95% CI, 0.63–0.70), 11% (HR, 0.89; 95% CI, 0.84–0.93), 10% (HR, 0.90; 95% CI, 0.86–0.95), and 10% (HR, 0.90; 95% CI, 0.85–0.94) lower risk of gout, respectively (Table 2).

In further analysis, the risks of gout decreased significantly with the increase of each 1 increment in healthy lifestyle factor (HR, 0.80, 95% CI, 0.77–0.83). In addition, an intermediate lifestyle and a favorable lifestyle were associated with a significantly lower risk of gout. Compared to an unfavorable lifestyle, the HRs (95% CIs) of gout were 0.79 (0.75–0.85) for an intermediate lifestyle and 0.63 (0.59–0.67) for a favorable lifestyle. Likewise, the Kaplan-Meier survival curve showed that an unfavorable lifestyle predicted the highest risk of gout (Additional file 1: Fig. S2b).

### Joint effect of lifestyle profile and genetic predisposition on gout risk

**Fig. 1:**
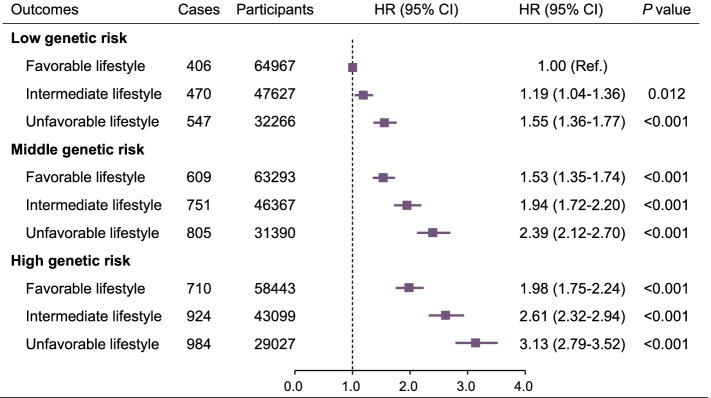
Multi-adjusted hazards ratios (HRs) and 95% confidence interval (CI) of gout by joint effect of lifestyle and genetic predisposition: results from Cox regression models. *Note*: Model adjusted for sex, age, socioeconomic status, education level, C-reactive protein, serum creatinine, cholesterol, triglyceride, cardiovascular disease, diabetes, hypertension, and BMI

Figure 1 shows the association between gout and the joint exposures of lifestyle profile and level of genetic predisposition. In joint effect analysis, compared to participants with low genetic predisposition and a favorable lifestyle, the HRs (95% CIs) of gout were 2.39 (2.12–2.70) and 3.12 (2.79–3.52) in those with middle and high genetic predisposition plus unfavorable lifestyle profiles, respectively, and 1.53 (1.35–1.74) and 1.98 (1.75–2.24) for those with middle and high genetic predisposition plus favorable lifestyle profiles, respectively (Additional file 1: Table S3). In addition, the reduced risk of gout associated with a favorable as opposed to an unfavorable lifestyle was 0.64 (95% CI, 0.56–0.73) among individuals with low genetic predisposition, 0.65 (95% CI, 0.58–0.72) among individuals with middle genetic predisposition, and 0.62 (95% CI, 0.57–0.69) among those with high genetic predisposition. There was a significant additive interaction between an unfavorable lifestyle and a high genetic risk of gout (RERI: 0.745, 95% CI: 0.441–1.049; AP: 0.223, 95% CI: 0.138–0.308; SI: 1.467, 95% CI: 1.227–1.755) (Additional file 1: Table S4). There was no significant multiplication interaction (*P*=0.779).

**Fig. 2:**
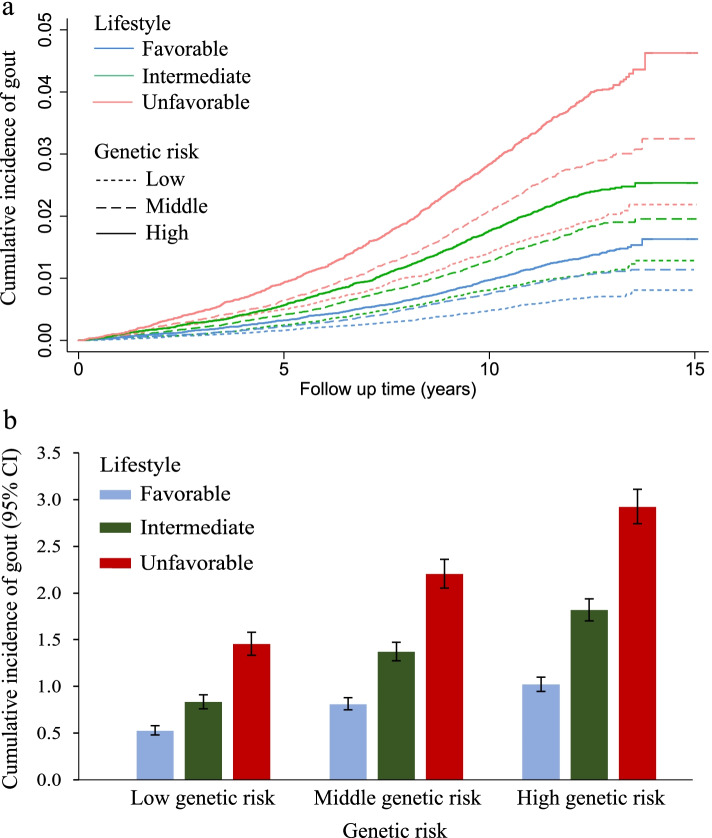
Incidence of gout by joint effect of lifestyle and genetic predisposition. **a** Cumulative incidence of gout during 15 years of follow-up by joint effect of lifestyle and genetic predisposition. **b** Cumulative incidence of gout per 1000 person-years at 15 years of follow-up by joint effect of lifestyle and genetic predisposition. Error bars represent 95% CI of estimated cumulative incidence

The Kaplan-Meier survival curve showed that the risk of incident gout was highest for those with high genetic predisposition and an unfavorable lifestyle (Fig. 2a). The cumulative incidence rate of gout per 1000 person-years was 2.92 (95% CI: 2.74–3.11) for participants with high genetic predisposition and an unfavorable lifestyle and 0.52 (95% CI: 0.48–0.58) for those with low genetic predisposition and a favorable lifestyle (Fig. 2b).

### Joint effect of lifestyle and genetic predisposition on gout risk according to CMD status

We investigated whether the associations between gout risk and the joint exposures of lifestyle profile and genetic predisposition varied according to CMD status (Table 3). The incident rate of gout per 1000 person-years for participants with high genetic predisposition and an unfavorable lifestyle was 1.35 (95% CI: 1.13–1.51) in individuals without CMDs and 4.45 (95% CI: 4.13–4.77) in those with CMDs. Furthermore, we observed significant multiplication (*P*=0.001) and additive interaction between CMDs and the joint exposures of lifestyle profile and genetic predisposition on the risk of gout (RERI: 2.211, 95% CI: 1.282–3.141; AP: 0.280, 95% CI: 0.179–0.381; SI: 1.471, 95% CI, 1.239–1.746) (Additional file 1: Table S5).

**Table 3:** Basic- and multi-adjusted hazards ratios (HRs) and 95% confidence interval (CIs) of gout by joint exposures of lifestyle and genetic risks stratified by cardiometabolic diseases (CMD): results from Cox regression models

Joint effect		Without CMD (*n*=228,494)			With CMD (*n*=187,987)		
Lifestyle	Genetic risk	IR (95% CI)^a^	Basic-adjusted HR (95% CI)^b^	Multi-adjusted HR (95% CI)^c^	IR (95% CI)^a^	Basic-adjusted HR (95% CI)^b^	Multi-adjusted HR (95% CI)^c^
Favorable	Low	0.23 (0.20–0.29)	1.00 (Ref.)	1.00 (Ref.)	0.96 (0.85–1.07)	1.00 (Ref.)	1.00 (Ref.)
Intermediate	Low	0.32 (0.27–0.41)	1.29 (0.98–1.70)	1.13 (0.86–1.49)	1.45 (1.31–1.61)	1.38 (1.19–1.61)	1.20 (1.03–1.39)
Unfavorable	Low	0.66 (0.58–0.83)	2.36 (1.82––3.06)	1.81 (1.39–2.35)	2.24 (2.03–2.48)	1.96 (1.69–2.28)	1.50 (1.29–1.74)
Favorable	Middle	0.38 (0.33–0.45)	1.63 (1.28–2.08)	1.64 (1.28–2.10)	1.44 (1.31–1.58)	1.50 (1.30–1.74)	1.49 (1.29–1.73)
Intermediate	Middle	0.61 (0.52–0.71)	2.35 (1.84–3.00)	2.07 (1.62–2.64)	2.32 (2.13–2.52)	2.24 (1.95–2.57)	1.92 (1.67–2.21)
Unfavorable	Middle	0.92 (0.80–1.08)	3.31 (2.59–4.23)	2.54 (1.98–3.25)	3.47 (3.20–3.75)	3.07 (2.68–3.52)	2.37 (2.06-2.72)
Favorable	High	0.50 (0.46–0.61)	2.19 (1.73–2.78)	2.20 (1.73–2.78)	1.79 (1.64–1.96)	1.89 (1.64–2.18)	1.90 (1.64–2.19)
Intermediate	High	0.79 (0.74–0.95)	3.23 (2.56–4.08)	2.75 (2.18–3.48)	3.07 (2.84–3.30)	2.99 (2.61–3.42)	2.59 (2.26–2.96)
Unfavorable	High	1.35 (1.13–1.51)	4.94 (3.91–6.23)	3.67 (2.90–4.65)	4.45 (4.13–4.77)	3.96 (3.47–4.52)	3.00 (2.62–3.43)

^a^Incidence rates are provided per 1000 person-years

^b^Adjusted for sex and age

^c^Adjusted for sex, age, socioeconomic status, education level, C-reactive protein, serum creatinine, cholesterol, triglyceride, and BMI

*Abbreviations*: *IR* incidence rate

### Additional analyses

Similar results were obtained when we (1) applied a weighted score for lifestyle factors (Additional file 1: Tables S6–S7), (2) performed stratified analysis by age (Additional file 1: Table S8) and sex (Additional file 1: Table S9), (3) excluded participants who died or developed incident gout within the first 3-year follow-up period (Additional file 1: Table S10), (4) excluded participants with diuretic antihypertensive drugs (Additional file 1: Table S11), and (5) repeated the analyses using a competing risk regression model (Additional file 1: Table S12).

## Discussion

In this large population-based prospective cohort study, we found that (1) genetic factors are associated with a higher risk of gout, (2) a healthy lifestyle is associated with a lower risk of gout, (3) gout risk can be mitigated by a favorable lifestyle in people with a middle to high genetic predisposition for gout, and (4) the association between gout and the joint exposures of lifestyle profile and genetic predisposition is mildly mitigated in individuals with CMDs compared to individuals without CMDs.

In recent years, polygenetic risk scores aggregating multiple risk alleles have been used to quantitatively measure genetic susceptibility to several polygenetic diseases, including dementia, cardiovascular disease, diabetes, and breast cancer [28, 35–39]. To date, one study has created a GRS for gout using urate-associated genetic variants, demonstrating that participants with a GRS greater than or equal to the mean had a nearly 3-fold increased risk of gout compared to those with a GRS less than the mean [40]. In the current study, a weighted genetic risk score of gout was calculated based on previous GWAS data [18]. We found that a high genetic risk score was associated with approximately 2-fold increased risk of gout compared to a low genetic risk score.

Emerging evidence has suggested that individual healthy lifestyle factors are associated with reduced gout risk [41–43]. However, few studies have investigated the association between an overall lifestyle profile and gout. In the current study, we found that a favorable lifestyle was related a lower risk of gout. From a public health perspective, our use of a simple scoring algorithm to define a lifestyle profile makes these epidemiological findings easier to interpret and translate into practice, therefore making them more informative for the general population. Further clinical trials on lifestyle interventions will be necessary to assess whether the observed associations are causal.

Several cross-sectional/case-cohort studies have pointed to a significant interaction between genetics and specific lifestyle factors in the context of gout risk. A case-control study by Rasheed et al. including 2729 European and Polynesian adults from New Zealand reported that alcohol exposure suppressed the gout risk conferred by the A-positive APOBEC1 complementation factor (*A1CF*) genotype. Furthermore, alcohol exposure eliminated the risk effect of glucokinase regulator (*GCKR*) on gout in people of European but not Polynesian origin [44]. Similarly, Rasheed et al. suggested that there was a non-additive interaction between alcohol consumption and lipoprotein receptor-related protein 2 (*LRP2*) on gout risk, where alcohol intake was associated with a 4.2-fold increased risk for individuals with the CC genotype, compared to a 1.1-fold increased risk for those with the CT or TT genotypes [45]. The Atherosclerosis Risk in Communities (ARIC) study suggested that *SLC2A9* genotype and sugar-sweetened beverage consumption interact to determine gout risk [46]. Moreover, a cross-sectional study conducted by Vicky et al. reported that the association between a urate GRS and gout is mildly attenuated in obese vs. overweight individuals [40]. We found that a healthy lifestyle can counteract a high genetic predisposition for gout and that individuals with a low genetic predisposition for gout can lose their inherent protection if they have an unfavorable lifestyle. To our knowledge, this is the first prospective study to assess the joint effect of lifestyle and genetic factors on gout risk.

We demonstrated that the association between gout and the joint exposures of lifestyle profile and genetic predisposition is moderately mitigated in individuals with vs. without CMDs. However, joint exposures of lifestyle profile and genetic predisposition have a strong effect on gout risk in those with and without CMDs. Accumulating evidence has suggested that lifestyle factors including alcohol consumption, smoking, diet, and physical activity are associated with CMD risk [47]. Likewise, a trans-ethnic GWAS meta-analysis based on 457,690 individuals found that serum urate showed significant genetic correlations with CMDs including diabetes, hypertension, heart disease, and stroke, with genetic causality analyses supporting a substantial role for pleiotropy [48]. The 7-fold increased risk of gout (HR 7.90) reported in the current study for individuals with a genetic predisposition for gout, an unfavorable lifestyle, and CMDs points to the clinical importance of preventing CMDs in people with gout.

The mechanisms underlying the interaction between the genetic risk of gout and an unfavorable lifestyle are multifactorial and incompletely understood. Like any other complex phenotype, gout results from the interplay between inherited genetic risk variants and environmental exposures. The SNPs that were selected to create the GRS for gout can be connected into functional or biochemical pathways, including renal and gut excretion of uric acid and the carbohydrate metabolic pathway, which includes the regulation of glycolysis, glucose, and insulin [18]. Available evidence has shown that lifestyle factors including alcohol consumption, smoking, physical activity, diet, and overweight are associated with metabolic disorders such as hyperlipidemia, metabolic syndrome, hypertension, and diabetes [49–52]. Rasheed et al. have also suggested that alcohol can impact gout risk through metabolic pathways in addition to or instead of directly interfering with renal or extrarenal uric acid excretion [44].

This study has several strengths, including a large study sample with a long follow-up period, the use of standardized protocols for data collection, and comprehensive diagnoses using multiple resources (e.g., physical examination, hospital inpatient records, and death registers). Despite these strengths, the study has several limitations that must be considered. First, potential changes in lifestyle factors after the baseline examination may have influenced our risk estimates. Second, we identified gout using medical records and cause of death during follow-up, and patients with well-controlled gout or minor attacks may not be recorded in the hospitalization records, resulting in underestimation of the incidence of gout. Additionally, people who volunteer for the UK Biobank cohort tend to be, on average, more health-conscious than nonparticipants, which may lead to underestimation incidence of gout [53]. Third, patients with diagnosed gout before the first date of UK biobank record may be regarded as incident cases without a proper observation period, and such misclassification is more likely to be non-differential and lead to an underestimation of the given association. Four, although all analyses in the present study were adjusted for known potential sources of bias, the possibility of unmeasured confounding factors and reverse causation remains. Finally, participants in the present study were primarily of white British descent, limiting the generalizability of the findings to Afro-British, Asian British, and other ethnic groups that may be at higher risk for gout.

## Conclusion

In this large-scale population-based cohort study, we found that genetic predisposition and unhealthy lifestyle factors were associated with increased risk of gout. However, a healthy lifestyle—including no/moderate alcohol consumption, non-smoking, regular physical activity, and a healthy diet—may attenuate the risk of gout related to genetic factors. The association between gout and the joint exposures of lifestyle profile and genetic predisposition is mildly mitigated in individuals with CMDs compared to individuals without CMDs. Our findings highlight the importance of maintaining a healthy lifestyle for the prevention of gout in people with a genetic predisposition.

## Supplementary Information

**Additional file 1: Methods**. Polygenic risk score. **Table S1**. Definition of lifestyle factors. **Table S2**. Single nucleotide polymorphisms (SNPs) used to build the polygenetic risk score for gout. **Table S3**. Basic- and multi-adjusted hazards ratios (HR) and 95% confidence interval (CI) of gout by joint exposures of lifestyle and genetic risks: results from Cox regression models. **Table S4**. Additive interaction between joint exposures of lifestyle and genetic risks for the risk of gout. **Table S5**. Additive interaction between joint exposures of lifestyle and genetic risks and cardiometabolic diseases (CMD) for the risk of gout. **Table S6**. Basic- and multi-adjusted hazards ratios (HRs) and 95% confidence interval (CIs) of gout by weight lifestyle score: results from Cox regression models. **Table S7**. Basic- and multi-adjusted hazards ratios (HRs) and 95% confidence interval (CIs) of gout by joint exposures of weight lifestyle score and genetic risks: results from Cox regression models. **Table S8**. Basic- and multi-adjusted hazards ratios (HRs) and 95% confidence interval (CIs) of gout by joint exposures of lifestyle and genetic risks by age: results from Cox regression models. **Table S9**. Basic- and multi-adjusted hazards ratios (HRs) and 95% confidence interval (CIs) of gout by joint exposures of lifestyle and genetic risks by sex: results from Cox regression models. **Table S10**. Basic- and multi-adjusted hazards ratios (HRs) and 95% confidence interval (CIs) of gout by joint exposures of lifestyle and genetic risks after excluding first 3 years incidence of gout or death during follow-up: results from Cox regression models. **Table S11**. Basic- and multi-adjusted hazards ratios (HRs) and 95% confidence interval (CIs) of gout by joint exposures of lifestyle and genetic risks after excluding participants with diuretic antihypertensive drugs at baseline: results from Cox regression models. **Table S12**. Basic- and multi-adjusted hazards ratios (HRs) and 95% confidence interval (CIs) of gout by joint exposures of lifestyle and genetic risks: results from Cox regression models: results from competing risk regression models. **Fig. S1**. Flowchart for the selection of the analyzed study sample from the UK Biobank Study. **Fig. S2**. Cumulative incidence of gout during follow-up.

## Data Availability

The data that support the findings of this study are available from UK Biobank (https://www.ukbiobank.ac.uk/), but restrictions apply to the availability of these data, which were used under license for the current study, and so are not publicly available. Data are however available from the authors upon reasonable request and with permission of UK Biobank.

## Abbreviations

A1CF: APOBEC1 complementation factor
AP: Attributable proportion
ARIC: Atherosclerosis Risk in Communities
BMI: Body mass index
CI: Confidence interval
CMD: Cardiometabolic disease
DBP: Diastolic blood pressure
GCKR: Glucokinase regulator
GRS: Polygenic risk score
GWAS: Genome-wide association study
HR: Hazard ratio
ICD: International Classification of Diseases
IPAQ: International Physical Activity Questionnaire
IR: Incidence rate
LRP2: Lipoprotein receptor-related protein 2
RERI: Relative excess risk due to interaction
SBP: Systolic blood pressure
SI: Synergy index
SNP: Single nucleotide polymorphism
