# Supplementary material for: Healthy lifestyle counteracts the risk effect of genetic factors on incident gout: a large population-based longitudinal study
Source: BMC Med. 2022 Apr 29;20:138. doi: 10.1186/s12916-022-02341-0 (PMC9052486; doi:10.1186/s12916-022-02341-0)
Supplement: Supplementary file 1 — Additional file 1: Methods. Polygenic risk score. Table S1. Definition of lifestyle factors. Table S2. Single nucleotide polymorphisms (SNPs) used to build the polygenetic risk score for gout. Table S3. Basic- and multi-adjusted hazards ratios (HR) and 95% confidence interval (CI) of gout by joint exposures of lifestyle and genetic risks: results from Cox regression models. Table S4. Additive interaction between joint exposures of lifestyle and genetic risks for the risk of gout. Table S5. Additive interaction between joint exposures of lifestyle and genetic risks and cardiometabolic diseases (CMD) for the risk of gout. Table S6. Basic- and multi-adjusted hazards ratios (HRs) and 95% confidence interval (CIs) of gout by weight lifestyle score: results from Cox regression models. Table S7. Basic- and multi-adjusted hazards ratios (HRs) and 95% confidence interval (CIs) of gout by joint exposures of weight lifestyle score and genetic risks: results from Cox regression models. Table S8. Basic- and multi-adjusted hazards ratios (HRs) and 95% confidence interval (CIs) of gout by joint exposures of lifestyle and genetic risks by age: results from Cox regression models. Table S9. Basic- and multi-adjusted hazards ratios (HRs) and 95% confidence interval (CIs) of gout by joint exposures of lifestyle and genetic risks by sex: results from Cox regression models. Table S10. Basic- and multi-adjusted hazards ratios (HRs) and 95% confidence interval (CIs) of gout by joint exposures of lifestyle and genetic risks after excluding first 3 years incidence of gout or death during follow-up: results from Cox regression models. Table S11. Basic- and multi-adjusted hazards ratios (HRs) and 95% confidence interval (CIs) of gout by joint exposures of lifestyle and genetic risks after excluding participants with diuretic antihypertensive drugs at baseline: results from Cox regression models. Table S12. Basic- and multi-adjusted hazards ratios (HRs) and 95% confidence interval (CIs) of gout [file 12916_2022_2341_MOESM1_ESM.doc]

**Additional File 1**

**Methods:** Polygenic risk score

**Table S1.** Definition of lifestyle factors

**Table S2.** Single nucleotide polymorphisms (SNPs) used to build the polygenetic risk score for gout

**Table S3.** Basic- and multi-adjusted hazards ratios (HR) and 95% confidence interval (CI) of gout by joint exposures of lifestyle and genetic risks: results from Cox regression models

**Table S4.** Additive interaction between joint exposures of lifestyle and genetic risks for the risk of gout

**Table S5.** Additive interaction between joint exposures of lifestyle and genetic risks and cardiometabolic diseases (CMD) for the risk of gout

**Table S6.** Basic- and multi-adjusted hazards ratios (HRs) and 95% confidence interval (CIs) of gout by weight lifestyle score: results from Cox regression models

**Table S7.** Basic- and multi-adjusted hazards ratios (HRs) and 95% confidence interval (CIs) of gout by joint exposures of weight lifestyle score and genetic risks: results from Cox regression models

**Table S8.** Basic- and multi-adjusted hazards ratios (HRs) and 95% confidence interval (CIs) of gout by joint exposures of lifestyle and genetic risks by age: results from Cox regression models

**Table S9.** Basic- and multi-adjusted hazards ratios (HRs) and 95% confidence interval (CIs) of gout by joint exposures of lifestyle and genetic risks by sex: results from Cox regression models

**Table S10.** Basic- and multi-adjusted hazards ratios (HRs) and 95% confidence interval (CIs) of gout by joint exposures of lifestyle and genetic risks after excluding first 3 years incidence of gout or death during follow-up: results from Cox regression models

**Table S11**. Basic- and multi-adjusted hazards ratios (HRs) and 95% confidence interval (CIs) of gout by joint exposures of lifestyle and genetic risks after excluding participants with diuretic antihypertensive drugs at baseline: results from Cox regression models

**Table S12.** Basic- and multi-adjusted hazards ratios (HRs) and 95% confidence interval (CIs) of gout by joint exposures of lifestyle and genetic risks: results from Cox regression models: results from competing risk regression models

**Fig. S1.** Flowchart for the selection of the analyzed study sample from the UK Biobank Study

**Fig. S2.** Cumulative incidence of gout during follow-up

**Methods:** **Genic risk score (GRS)**

To generate a genic risk score (GRS), each single nucleotide polymorphism (SNP) was recoded as 0, 1, or 2 according to the number of risk-increasing alleles. The score was calculated using the equation:

Score=β_1_×SNP_1_+β_2_×SNP_2_+…+β_n-1_×SNP_n-1_ +…+β_n_×SNP_n_

where n is the total number of SNPs, and β is per-allele log odds ratio (OR) for associated with SNP_n_. The effect size estimates of β were taken from a genome wide association study that carried out in subjects from Global Urate Genetics Consortium study of individuals of European ancestry. GRS were categorized as low (lowest tertile), middle (tertile 2) and high (highest tertile).

**Table S1. Definition of lifestyle factors**

| Factors | Unhealthy level | Healthy level |
| --- | --- | --- |
| Alcohol consumption | Excessive consumption: women: >14g/day  men: >28g/day | Never or moderate consumption:  women: 0 and ≤14g/day  men: 0 and ≤28g/day  or never deinked |
| Smoking status | Smoking | Non-smoking |
| Physical activity | 0-149 min/week of moderate activity OR 0-74 min/week of vigorous activity OR 0-149 min/week of moderate and vigorous activity | ≥150 min/week of moderate activity OR ≥75 min/week of vigorous activity OR ≥150 min/week of moderate and vigorous activity |
| Diet | Intake of 0-3 healthy dietary components:  1. Fruits: ≥ 3 servings/day  2. Vegetables: ≥ 3 servings/day  3. Fish: ≥2 servings/week  4. Processed meats: ≤ 1 serving/week  5.Unprocessed red meats: ≤1.5 servings/week  6. Whole grains: ≥ 3servings/day  7. Refined grains: ≤1.5 servings/day | Intake of ≥ 4 dietary healthy dietary components:  1. Fruits: ≥ 3 servings/day  2. Vegetables: ≥ 3 servings/day  3. Fish: ≥2 servings/week  4. Processed meats: ≤ 1 serving/week  5.Unprocessed red meats: ≤1.5 servings/week  6. Whole grains: ≥ 3servings/day  7. Refined grains: ≤1.5 servings/day |

**Table S2. Single nucleotide polymorphisms (SNPs) used to build the polygenetic risk score for gout**

| SNPs | Chr. | Position | Closest gene | A2 | A1 | MAF | Beta | P value |
| --- | --- | --- | --- | --- | --- | --- | --- | --- |
| rs11590889 | 1 | 8127916 | PARK7 | C | T | 0.43 | -0.14 | 6.27E-05 |
| rs2799541 | 1 | 92355115 | TGFBR3 | G | A | 0.02 | 0.74 | 4.15E-05 |
| rs12564416 | 1 | 201345072 | TNNT2 | G | A | 0.02 | 0.49 | 4.22E-05 |
| rs2794271 | 1 | 232133186 | DISC1 | G | A | 0.01 | 1.6 | 1.43E-05 |
| rs1260326 | 2 | 27584444 | GCKR | C | T | 0.41 | 0.14 | 3.57E-05 |
| rs10208230 | 2 | 80173393 | CTNNA2 | C | T | 0.11 | 0.11 | 9.23E-05 |
| rs2080312 | 2 | 102774810 | IL1R1 | G | A | 0.02 | -1 | 2.13E-05 |
| rs7644206 | 3 | 36571784 | STAC | G | A | 0.14 | -0.21 | 2.12E-05 |
| rs12498742 | 4 | 9553150 | SLC2A9 | G | A | 0.77 | 0.45 | 2.42E-25 |
| rs4604059 | 4 | 10115065 | WDR1 | C | T | 0.47 | -0.25 | 9.62E-14 |
| rs2231142 | 4 | 89271347 | ABCG2 | G | T | 0.11 | 0.54 | 4.98E-32 |
| rs2158098 | 5 | 11380161 | CTNND2 | C | T | 0.4 | 0.15 | 3.26E-05 |
| rs17170910 | 5 | 136399774 | SPOCK1 | G | A | 0.02 | -0.51 | 3.15E-05 |
| rs1165151 | 6 | 25929595 | SLC17A1 | G | T | 0.47 | -0.15 | 5.30E-07 |
| rs729761 | 6 | 43912549 | VEGFA | G | T | 0.3 | -0.13 | 6.45E-05 |
| rs6972511 | 7 | 78091663 | MAGI2 | G | A | 0.14 | 0.14 | 7.09E-05 |
| rs16933301 | 8 | 68344106 | ARFGEF1 | G | A | 0.02 | 0.49 | 4.06E-05 |
| rs13287980 | 9 | 106364021 | NIPSNAP3A | G | A | 0.24 | 0.24 | 9.71E-07 |
| rs12098803 | 10 | 11280099 | CELF2 | C | T | 0.05 | 0.62 | 2.22E-05 |
| rs11257912 | 10 | 12638108 | CAMK1D | G | A | 0.49 | 0.14 | 5.14E-05 |
| rs951202 | 10 | 84048536 | NRG3 | C | T | 0.29 | -1.9 | 1.21E-05 |
| rs11245316 | 10 | 126298185 | LHPP | G | T | 0.21 | 0.18 | 2.11E-05 |
| rs11605578 | 11 | 21063299 | NELL1 | G | C | 0.18 | 0.2 | 6.78E-06 |
| rs2078267 | 11 | 64090690 | SLC22A11 | C | T | 0.51 | -0.15 | 6.02E-06 |
| rs7123206 | 11 | 88951332 | TYR | C | T | 0.12 | 0.12 | 9.23E-05 |
| rs11172147 | 12 | 57696677 | R3HDM2 | G | A | 0.19 | -0.17 | 1.38E-05 |
| rs11837061 | 12 | 106106151 | CASC18 | C | A | 0.11 | 0.11 | 7.12E-05 |
| rs12324861 | 15 | 58049540 | POLR2M | G | A | 0.01 | 1.1 | 2.16E-06 |
| rs4965299 | 15 | 100806546 | ADAMTS17 | G | A | 0.28 | 0.14 | 2.88E-05 |
| rs1064875 | 16 | 3102209 | MMP25 | G | A | 0.18 | 0.19 | 7.57E-06 |
| rs8075363 | 17 | 65643972 | PITPNC1 | G | A | 0.04 | 0.37 | 1.35E-05 |
| rs16995525 | 20 | 15289701 | MACROD2 | G | A | 0.09 | 0.09 | 7.66E-05 |
| rs6142830 | 20 | 60262743 | CDH4 | C | T | 0.13 | 0.2 | 1.44E-05 |

**Table S3. Basic- and multi-adjusted hazards ratios (HR) and 95% confidence interval (CI) of gout by joint exposures of lifestyle and genetic risks: results from Cox regression models**

| Joint effect | | IR (95% CI)^a^ | Basic-adjusted  HR (95% CI)^b^ | Multi-adjusted  HR (95% CI)^c^ |
| --- | --- | --- | --- | --- |
| Lifestyle | Genetic risk |
| Favorable | Low | 0.52 (0.48-0.58) | 1.00 (Ref.) | 1.00 (Ref.) |
| Intermediate | Low | 0.83 (0.76-0.91) | 1.41 (1.24-1.62) | 1.19 (1.04-1.36) |
| Unfavorable | Low | 1.45 (1.33-1.58) | 2.17 (1.91-2.47) | 1.55 (1.36-1.77) |
| Favorable | Middle | 0.81 (0.75-0.88) | 1.53 (1.35-1.74) | 1.53 (1.35-1.74) |
| Intermediate | Middle | 1.37 (1.28-1.47) | 2.35 (2.08-2.65) | 1.94 (1.72-2.20) |
| Unfavorable | Middle | 2.20 (2.05-2.36) | 3.32 (2.95-3.74) | 2.39 (2.12-2.70) |
| Favorable | High | 1.02 (0.95-1.10) | 1.96 (1.73-2.21) | 1.98 (1.75-2.24) |
| Intermediate | High | 1.82 (1.70-1.94) | 3.14 (2.79-3.53) | 2.61 (2.32-2.94) |
| Unfavorable | High | 2.92 (2.74-3.11) | 4.45 (3.96-4.99) | 3.13 (2.79-3.52) |

^a^Incidence rates are provided per 1000 person-years.

^b^Adjusted for sex and age.

^c^Adjusted for sex, age, socioeconomic status, education level, C-reactive protein, serum creatinine, cholesterol, triglyceride, cardiovascular disease, diabetes, hypertension, and BMI.

Abbreviations: CI, confidence interval; HR, hazard ratio; IR, incidence rate.

**Table S4. Additive interaction between lifestyle and genetic risk for the risk of gout**

| Joint exposure | | IR (95% CI)^a^ | Basic-adjusted  HR (95% CI)^b^ | Multi-adjusted  HR (95% CI)^c^ |
| --- | --- | --- | --- | --- |
| Lifestyle | genetic risk |
| Favorable | Low | 0.52 (0.48-0.58) | 1.00 (Ref.) | 1.00 (Ref.) |
| Favorable | High | 1.02 (0.95-1.10) | 1.98 (1.75-2.24) | 2.00 (1.77-2.27) |
| Unfavorable | Low | 1.45 (1.33-1.58) | 2.13 (1.87-2.42) | 1.59 (1.39-1.81) |
| Unfavorable | High | 2.92 (2.74-3.11) | 4.44 (3.95-5.00) | 3.34 (2.96-3.77) |

^a^Incidence rates are provided per 1000 person-years.

^b^Adjusted for sex, age.

^c^Adjusted for sex, age, socioeconomic status, education level, C-reactive protein, serum creatinine, cholesterol, triglyceride, cardiovascular disease, diabetes, hypertension, and BMI.

Abbreviations: IR, incidence rate.

**Measures of additive interaction for gout:**

Relative excess risk due to interaction (RERI): 0.745, 95% CI: 0.441, 1.049

Attributable proportion due to interaction (AP): 0.223, 95% CI: 0.138, 0.308

Synergy index (SI): 1.467, 95% CI: 1.227, 1.755.

**Table S5. Additive interaction between joint exposures of lifestyle and genetic risks and cardiometabolic diseases (CMD) for the risk of gout**

| Joint exposure | | IR (95% CI)^a^ | Basic-adjusted  HR (95% CI)^b^ | Multi-adjusted  HR (95% CI)^c^ |
| --- | --- | --- | --- | --- |
| Lifestyle/genetic risk | CMD |
| Favorable/low | No | 0.23 (0.20-0.29) | 1.00 (Ref.) | 1.00 (Ref.) |
| Favorable/low | Yes | 0.96 (0.85-1.07) | 5.06 (4.00-6.40) | 4.34 (3.43-5.49) |
| Unfavorable/high | No | 1.35 (1.13-1.51) | 2.65 (2.11-3.32) | 2.35 (1.87-2.95) |
| Unfavorable/high | Yes | 4.45 (4.13-4.77) | 10.39 (8.42-12.81) | 7.90 (6.39-9.78) |

^a^Incidence rates are provided per 1000 person-years.

^b^Adjusted for sex and age.

^c^Adjusted for sex, age, socioeconomic status, education level, C-reactive protein, serum creatinine, cholesterol, triglyceride, and BMI.

Abbreviations: IR, incidence rate.

**Measures of additive interaction for gout:**

Relative excess risk due to interaction (RERI): 2.211, 95% CI: 1.282, 3.141

Attributable proportion due to interaction (AP): 0.280, 95% CI: 0.178, 0.381

Synergy index (SI): 1.471, 95% CI: 1.239, 1.746.

**Table S6. Basic- and multi-adjusted hazards ratios (HRs) and 95% confidence interval (CIs) of gout by weighted lifestyle score: results from Cox regression model**

| Weighted lifestyle | IR (95% CI)^a^ | Basic-adjusted  HR (95% CI)^b^ | Multi-adjusted  HR (95% CI)^c^ |
| --- | --- | --- | --- |
| Unfavorable | 1.88 (1.82-1.95) | 1.00 (Ref.) | 1.00 (Ref.) |
| Intermediate | 1.37 (1.27-1.47) | 0.73 (0.67-0.79) | 0.74 (0.67-0.80) |
| Favorable | 0.99 (0.95-1.02) | 0.53 (0.50-0.56) | 0.62 (0.59-0.66) |

^a^Incidence rates are provided per 1000 person-years.

^b^Adjusted for sex and age.

^c^Adjusted for sex, age, socioeconomic status, education level, C-reactive protein, serum creatinine, cholesterol, triglyceride, cardiovascular disease, diabetes, hypertension, and BMI.

Abbreviations: IR, incidence rate.

**Table S7. Basic- and multi-adjusted hazards ratios (HRs) and 95% confidence interval (CIs) of gout by joint exposures of weighted lifestyle score and genetic risks: results from Cox regression models**

| Joint effect | | IR (95% CI)^a^ | Basic-adjusted  HR (95% CI)^b^ | Multi-adjusted  HR (95% CI)^c^ |
| --- | --- | --- | --- | --- |
| Lifestyle | Genetic risk |
| Favorable | Low | 0.62 (0.58-0.68) | 1.00 (Ref.) | 1.00 (Ref.) |
| Intermediate | Low | 0.97 (0.84-1.13) | 1.50 (1.26-1.79) | 1.29 (1.08-1.53) |
| Unfavorable | Low | 1.23 (1.14-1.33) | 1.91 (1.71-2.13) | 1.62 (1.45-1.81) |
| Favorable | Middle | 1.02 (0.95-1.08) | 1.60 (1.44-1.77) | 1.58 (1.43-1.75) |
| Intermediate | Middle | 1.30 (1.14-1.48) | 2.07 (1.77-2.42) | 1.77 (1.51-2.07) |
| Unfavorable | Middle | 1.92 (1.81-2.04) | 3.04 (2.75-3.37) | 2.60 (2.35-2.88) |
| Favorable | High | 1.34 (1.27-1.43) | 2.11 (1.91-2.34) | 2.12 (1.92-2.34) |
| Intermediate | High | 1.86 (1.66-2.10) | 2.90 (2.51-3.35) | 2.49 (2.16-2.88) |
| Unfavorable | High | 2.54 (2.42-2.70) | 3.97 (3.60-4.38) | 3.33 (3.01-3.67) |

^a^Incidence rates are provided per 1000 person-years.

^b^Adjusted for sex and age.

^c^Adjusted for sex, age, socioeconomic status, education level, C-reactive protein, serum creatinine, cholesterol, triglyceride, cardiovascular disease, diabetes, hypertension, and BMI.

Abbreviations: IR, incidence rate.

**Table S8. Basic- and multi-adjusted hazards ratios (HRs) and 95% confidence interval (CIs) of gout by joint exposures of lifestyle and genetic risks by age: results from Cox regression models**

| Joint effect | | Age <60 years (n=231,792) | | |  | Age ≥60 years (n=179,449) | | |
| --- | --- | --- | --- | --- | --- | --- | --- | --- |
| Lifestyle | Genetic risk | IR (95% CI)^a^ | Basic-adjusted  HR (95% CI)^b^ | Multi-adjusted  HR (95% CI)^c^ |  | IR (95% CI)^a^ | Basic-adjusted  HR (95% CI)^b^ | Multi-adjusted  HR (95% CI)^c^ |
| Favorable | Low | 0.30 (0.27-0.32) | 1.00 (Ref.) | 1.00 (Ref.) |  | 0.83 (0.75-0.90) | 1.00 (Ref.) | 1.00 (Ref.) |
| Intermediate | Low | 0.42 (0.38-0.45) | 1.26 (0.99-1.60) | 1.02 (0.80-1.30) |  | 1.39 (1.26-1.50) | 1.50 (1.28-1.76) | 1.28 (1.09-1.51) |
| Unfavorable | Low | 0.82 (0.75-0.89) | 2.20 (1.75-2.75) | 1.53 (1.22-1.92) |  | 2.27 (2.06-2.45) | 2.18 (1.87-2.55) | 1.55 (1.32-1.81) |
| Favorable | Middle | 0.48 (0.44-0.52) | 1.61 (1.29-2.00) | 1.57 (1.26-1.96) |  | 1.25 (1.14-1.35) | 1.50 (1.29-1.75) | 1.51 (1.29-1.76) |
| Intermediate | Middle | 0.77 (0.70-0.83) | 2.36 (1.91-2.92) | 1.91 (1.54-2.36) |  | 2.17 (1.98-2.35) | 2.36 (2.03-2.73) | 2.00 (1.73-2.32) |
| Unfavorable | Middle | 1.28 (1.17-1.39) | 3.42 (2.78-4.22) | 2.38 (1.93-2.93) |  | 3.40 (3.09-3.67) | 3.31 (2.86-3.82) | 2.43 (2.09-2.81) |
| Favorable | High | 0.61 (0.56-0.66) | 2.06 (1.67-2.54) | 2.06 (1.67-2.54) |  | 1.58 (1.44-1.71) | 1.91 (1.65-2.22) | 1.95 (1.68-2.26) |
| Intermediate | High | 1.10 (1.00-1.19) | 3.35 (2.73-4.09) | 2.73 (2.23-3.35) |  | 2.79 (2.54-3.01) | 3.05 (2.65-3.52) | 2.58 (2.24-2.98) |
| Unfavorable | High | 1.67 (1.52-1.80) | 4.52 (3.69-5.53) | 3.05 (2.49-3.75) |  | 4.56 (4.15-4.92) | 4.45 (3.87-5.13) | 3.23 (2.80-3.72) |

^a^Incidence rates are provided per 1000 person-years.

^b^Adjusted for sex.

^c^Adjusted for sex, socioeconomic status, education level, C-reactive protein, serum creatinine, cholesterol, triglyceride, cardiovascular disease, diabetes, hypertension, and BMI.

Abbreviations: IR, incidence rate.

**Table S9. Basic- and multi-adjusted hazards ratios (HRs) and 95% confidence interval (CIs) of gout by joint exposures of lifestyle and genetic risks by sex: results from Cox regression models**

| Joint effect | | Women (n=221,914) | | |  | Men (n=189,327) | | |
| --- | --- | --- | --- | --- | --- | --- | --- | --- |
| Lifestyle | Genetic risk | IR (95% CI)^a^ | Basic-adjusted  HR (95% CI)^b^ | Multi-adjusted  HR (95% CI)^c^ |  | IR (95% CI)^a^ | Basic-adjusted  HR (95% CI)^b^ | Multi-adjusted  HR (95% CI)^c^ |
| Favorable | Low | 0.18 (0.14-0.22) | 1.00 (Ref.) | 1.00 (Ref.) |  | 1.03 (0.94-1.11) | 1.00 (Ref.) | 1.00 (Ref.) |
| Intermediate | Low | 0.29 (0.23-0.36) | 1.66 (1.23-2.24) | 1.38 (1.02-1.87) |  | 1.46 (1.33-1.58) | 1.38 (1.19-1.60) | 1.17 (1.01-1.36) |
| Unfavorable | Low | 0.40 (0.31-0.50) | 2.38 (1.73-3.28) | 1.68 (1.21-2.32) |  | 2.33 (2.12-2.52) | 2.17 (1.88-2.49) | 1.58 (1.37-1.83) |
| Favorable | Middle | 0.27 (0.23-0.32) | 1.49 (1.13-1.97) | 1.61 (1.21-2.14) |  | 1.60 (1.46-1.73) | 1.55 (1.34-1.78) | 1.54 (1.34-1.78) |
| Intermediate | Middle | 0.36 (0.29-0.43) | 2.10 (1.58-2.80) | 1.77 (1.32-2.36) |  | 2.55 (2.32-2.76) | 2.41 (2.11-2.76) | 2.02 (1.76-2.31) |
| Unfavorable | Middle | 0.51 (0.41-0.63) | 3.12 (2.31-4.21) | 2.17 (1.60-2.95) |  | 3.62 (3.29-3.91) | 3.38 (2.96-3.86) | 2.49 (2.18-2.84) |
| Favorable | High | 0.36 (0.30-0.42) | 1.96 (1.49-2.56) | 2.12 (1.61-2.79) |  | 2.00 (1.82-2.16) | 1.96 (1.71-2.24) | 1.98 (1.72-2.27) |
| Intermediate | High | 0.50 (0.42-0.59) | 2.75 (2.09-3.62) | 2.30 (1.74-3.05) |  | 3.40 (3.10-3.68) | 3.23 (2.84-3.68) | 2.73 (2.39-3.10) |
| Unfavorable | High | 0.82 (0.68-0.97) | 4.85 (3.68-6.39) | 3.24 (2.44-4.30) |  | 4.74 (4.31-5.11) | 4.43 (3.90-5.04) | 3.20 (2.81-3.64) |

^a^Incidence rates are provided per 1000 person-years.

^b^Adjusted for age.

^c^Adjusted for age, socioeconomic status, education level, C-reactive protein, serum creatinine, cholesterol, triglyceride, cardiovascular disease, diabetes, hypertension, and BMI.

Abbreviations: IR, incidence rate.

**Table S10. Basic- and multi-adjusted hazards ratios (HRs) and 95% confidence interval (CIs) of gout by joint exposures of lifestyle and genetic risks after excluding first 3 years incidence of gout or death during follow-up: results from Cox regression models**

| Joint effect | | IR (95% CI)^a^ | Basic-adjusted  HR (95% CI)^b^ | Multi-adjusted  HR (95% CI)^c^ |
| --- | --- | --- | --- | --- |
| Lifestyle | Genetic risk |
| Favorable | Low | 0.47 (0.42-0.52) | 1.00 (Ref.) | 1.00 (Ref.) |
| Intermediate | Low | 0.75 (0.68-0.83) | 1.44 (1.25-1.66) | 1.22 (1.06-1.40) |
| Unfavorable | Low | 1.24 (1.13-1.36) | 2.10 (1.83-2.41) | 1.51 (1.32-1.74) |
| Favorable | Middle | 0.73 (0.67-0.79) | 1.55 (1.36-1.78) | 1.55 (1.36-1.78) |
| Intermediate | Middle | 1.20 (1.12-1.30) | 2.33 (2.05-2.64) | 1.94 (1.70-2.20) |
| Unfavorable | Middle | 1.94 (1.80-2.09) | 3.31 (2.92-3.76) | 2.40 (2.11-2.73) |
| Favorable | High | 0.90 (0.83-0.97) | 1.94 (1.70-2.20) | 1.96 (1.72-2.23) |
| Intermediate | High | 1.58 (1.47-1.69) | 3.08 (2.72-3.48) | 2.57 (2.27-2.92) |
| Unfavorable | High | 2.58 (2.41-2.76) | 4.45 (3.93-5.03) | 3.17 (2.80-3.59) |

^a^Incidence rates are provided per 1000 person-years.

^b^Adjusted for sex and age.

^c^Adjusted for sex, age, socioeconomic status, education level, C-reactive protein, serum creatinine, cholesterol, triglyceride, cardiovascular disease, diabetes, hypertension, and BMI.

Abbreviations: IR, incidence rate.

**Table S11. Basic- and multi-adjusted hazards ratios (HRs) and 95% confidence interval (CIs) of gout by joint exposures of lifestyle and genetic risks after excluding participants with diuretic antihypertensive drugs at baseline: results from Cox regression models**

| Joint effect | | IR (95% CI)^a^ | Basic-adjusted  HR (95% CI)^b^ | Multi-adjusted  HR (95% CI)^c^ |
| --- | --- | --- | --- | --- |
| Lifestyle | Genetic risk |
| Favorable | Low | 0.50 (0.45-0.55) | 1.00 (Ref.) | 1.00 (Ref.) |
| Intermediate | Low | 0.79 (0.71-0.86) | 1.40 (1.22-1.60) | 1.18 (1.03-1.36) |
| Unfavorable | Low | 1.37 (1.25-1.49) | 2.14 (1.87-2.44) | 1.55 (1.36-1.77) |
| Favorable | Middle | 0.77 (0.71-0.84) | 1.53 (1.35-1.74) | 1.53 (1.34-1.74) |
| Intermediate | Middle | 1.30 (1.20-1.40) | 2.33 (2.05-2.63) | 1.93 (1.71-2.19) |
| Unfavorable | Middle | 2.09 (1.94-2.25) | 3.29 (2.91-3.72) | 2.40 (2.12-2.71) |
| Favorable | High | 0.97 (0.90-1.05) | 1.95 (1.72-2.21) | 1.96 (1.73-2.23) |
| Intermediate | High | 1.73 (1.62-1.85) | 3.13 (2.78-3.53) | 2.62 (2.32-2.96) |
| Unfavorable | High | 2.78 (2.60-2.96) | 4.42 (3.93-4.98) | 3.14 (2.79-3.55) |

^a^Incidence rates are provided per 1000 person-years.

^b^Adjusted for sex and age.

^c^Adjusted for sex, age, socioeconomic status, education level, C-reactive protein, serum creatinine, cholesterol, triglyceride, cardiovascular disease, diabetes, hypertension, and BMI.

Abbreviations: IR, incidence rate.

**Table S12. Basic- and multi-adjusted hazards ratios (HRs) and 95% confidence interval (CIs) of gout by joint exposures of lifestyle and genetic risks: results from competing risk regression model**

| Joint effect | | No. of  event | No. of  death | Basic-adjusted  HR (95% CI)^a^ | Multi-adjusted  HR (95% CI)^b^ |
| --- | --- | --- | --- | --- | --- |
| Lifestyle | Genetic risk |
| Favorable | Low | 406 | 3213 | 1.00 (Ref.) | 1.00 (Ref.) |
| Intermediate | Low | 470 | 3204 | 1.38 (1.21-1.58) | 1.20 (1.05-1.37) |
| Unfavorable | Low | 547 | 3197 | 2.11 (1.86-2.39) | 1.58 (1.39-1.79) |
| Favorable | Middle | 609 | 2986 | 1.53 (1.35-1.73) | 1.53 (1.35-1.73) |
| Intermediate | Middle | 751 | 3094 | 2.32 (2.06-2.61) | 1.97 (1.75-2.23) |
| Unfavorable | Middle | 805 | 3103 | 3.16 (2.81-3.56) | 2.37 (2.10-2.67) |
| Favorable | High | 710 | 2683 | 2.00 (1.78-2.26) | 2.01 (1.79-2.27) |
| Intermediate | High | 924 | 2786 | 3.08 (2.75-3.46) | 2.64 (2.35-2.96) |
| Unfavorable | High | 984 | 2818 | 4.41 (3.94-4.94) | 3.15 (2.90-3.65) |

^a^Adjusted for sex and age.

^b^Adjusted for sex, age, socioeconomic status, education level, C-reactive protein, serum creatinine, cholesterol, triglyceride, cardiovascular disease, diabetes, hypertension, and BMI.

Abbreviations: IR, incidence rate.


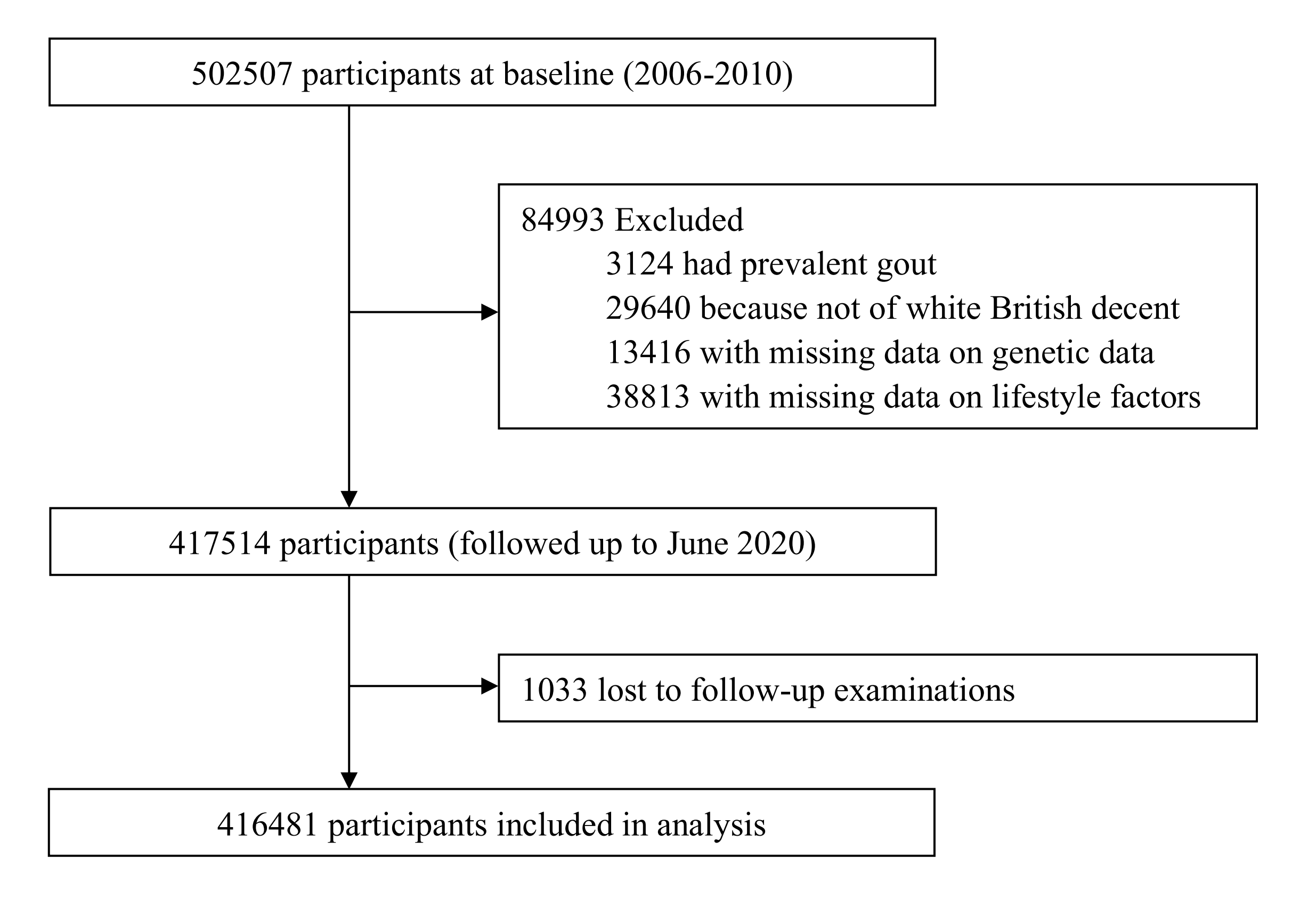


**Fig. S1. Flowchart for the selection of the analyzed study sample from the UK Biobank Study**


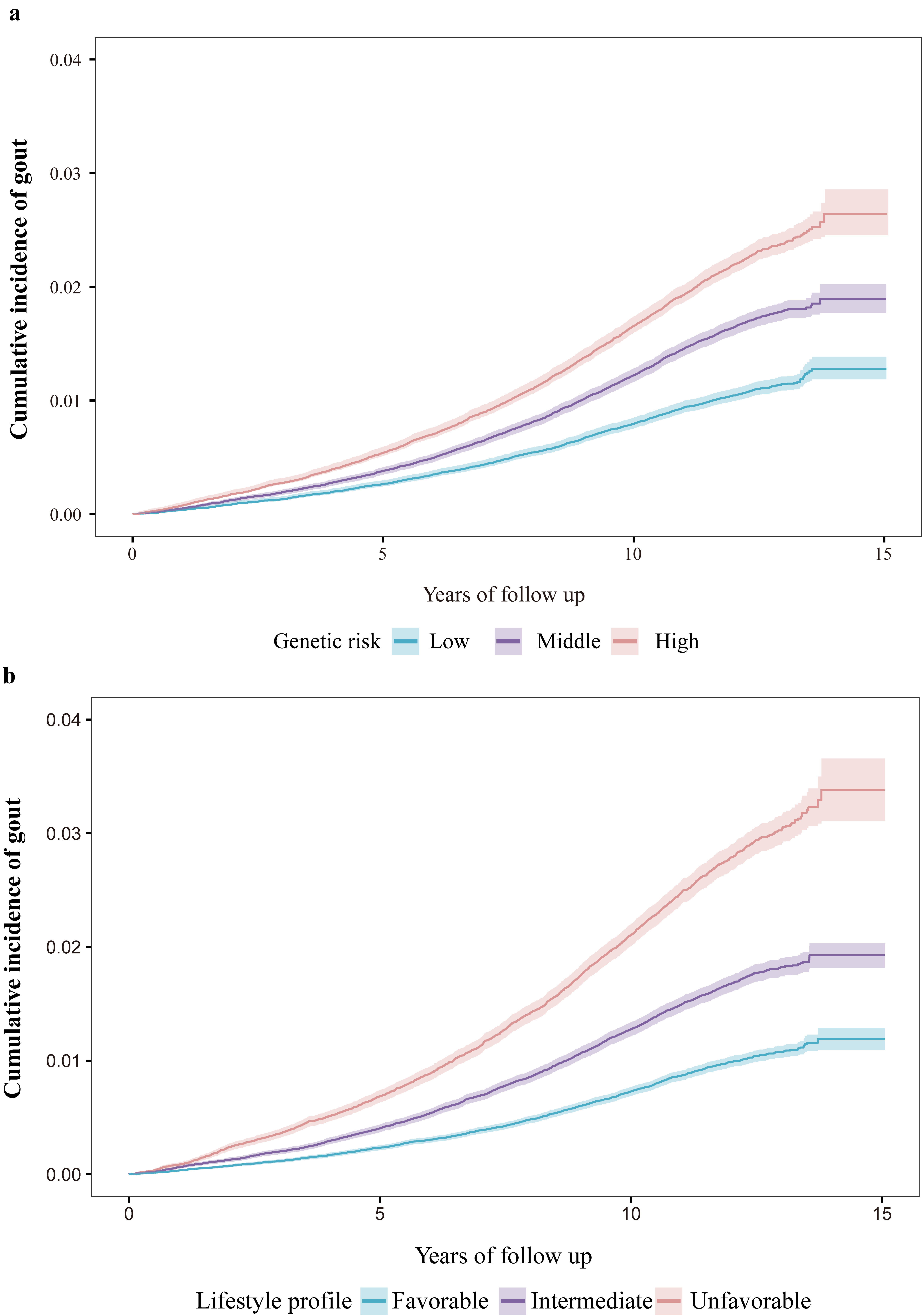


**Fig. S2. Cumulative incidence of gout during follow-up.**

Cumulative incidence in groups stratified according to genetic risk (a) and according to lifestyle profiles (b). For a and b, shaded areas represent 95% CI.
